# Practice of Medicine

**Published:** 1893-02

**Authors:** 


PRACTICE OF MEDICINE.—A Manual for Students and Practitioners; by Edwin T. Doubleday, M. D., Member New York Pathological Society, and J. D. Nagle, M. D., Member New York County Medical Association. Philadelphia, Lea Brothers & Co. Price $1.00

THE task of condensing the science of the practice of medicine into a manual, selecting that which is best, and which will be of most service to the student or practitioner, is one which requires a great amount of judgment and careful preparation. The author seems to have done his work well. C. E. J.

